# Supplementary material for: Hsa_circ_0015278 Regulates FLT3-ITD AML Progression via Ferroptosis-Related Genes
Source: Cancers (Basel). 2022 Dec 22;15(1):71. doi: 10.3390/cancers15010071 (PMC9817690; doi:10.3390/cancers15010071)
Supplement: Supplementary file 1 [file cancers-15-00071-s001.zip › Table S1. The detailed information of microarray datasets from GEO database.pdf]

Table S1. The detailed information of microarray datasets from GEO database

Data source	series	Platform	Author	Year	Samples (T&N)	Tissue
circRNA	GSE94591	GPL19978	Li W	2017	6/4	Bone marrow
mRNA	GSE65409	GPL6947	Tan SF	2015	30/8	PBMC
