# Supplementary material for: Hsa_circ_0015278 Regulates FLT3-ITD AML Progression via Ferroptosis-Related Genes
Source: Cancers (Basel). 2022 Dec 22;15(1):71. doi: 10.3390/cancers15010071 (PMC9817690; doi:10.3390/cancers15010071)
Supplement: Supplementary file 1 [file cancers-15-00071-s001.zip › Table S2. The sequences of primer.pdf]

Table S2. Primers of circRNA and mRNAs in present study

circRNAs/mRNA	Primer (5′- 3′)
hsa_circ_0015278	
divergent F	5′- CCTCAGGAAAACAGATGGCAC -3′
divergent R	5′- CAACCACCAATCCACTACGG -3′.
hsa_circ_0049365	
divergent F	5′- CTGTGGTGGTCTTGGTGAAG -3′
divergent R	5′- ACTTCTTGGCGTGGAGGTAG -3′
hsa_circ_0003162	
divergent F	5′- TGGTGGTGTAAAAGTGTGAAAGA -3′
divergent R	5′- GGTATCCCATAAAGCTACCCAC -3′
hsa_circ_0011536	
divergent F	5′- TGACAATGCTCAAGTGGTGG -3′
divergent R	5′- TGCAAGATCCCCAGAGTTCA -3′
GAPDH	
convergent F	5′- CATGTTCGTCATGGGTGTGAA -3′
convergent R	5′- GGCATGGACTGTGGTCATGAG -3′
