# Supplementary material for: Hsa_circ_0015278 Regulates FLT3-ITD AML Progression via Ferroptosis-Related Genes
Source: Cancers (Basel). 2022 Dec 22;15(1):71. doi: 10.3390/cancers15010071 (PMC9817690; doi:10.3390/cancers15010071)
Supplement: Supplementary file 1 [file cancers-15-00071-s001.zip › Table S3. Bioinformatics tools and databases.pdf]

Table S3 Bioinformatics tools and databases used in this study

Databse	Hyperlinks	Function
TCGA	https://portal.gdc.cancer.gov/	TCGA and GTEx were used to retrieve AML RNA-Seq and corresponding clinicopathological data.
GTEx	https://gtexportal.org/home/datasets	
Gene Expression Omnibus (GEO)	http://www.ncbi.nlm.nih.gov/geo	GEO was used to obtain circRNA and gene expression profiles microarray data.
FerrDb V2 website	http://www.zhounan.org/ferrdb/current/	The ferroptosis genes (Ferr-Genes) were downloaded from FerrDb V2 website.
CircInteractome	https://circinteractome.nia.nih.gov/	These website tools were used to predict the target miRNAs of circRNAs.
circbank	http://www.circbank.cn/index.html	
circAtlas	http://circatlas.biols.ac.cn/	
TargetScan	https://www.targetscan.org/vert_80/	These website tools were used to predict target mRNAs of the miRNAs.
ENCORI	https://starbase.sysu.edu.cn/	
miRWalk	http://mirwalk.umm.uni-heidelberg.de/	
Search Tool for the Retrieval of Interacting Genes (STRING)	https://cn.string-db.org/	The PPI network was built utilizing STRING.
lncLocator	http://www.csbio.sjtu.edu.cn/bioinf/lncLocator/index.html	The subcellular localization of circRNA was performed through the lncLocator online website.
Cancer-Specific CircRNA Database (CSCD)	http://gb.whu.edu.cn/CSCD/	CSCD was used to present the structural patterns of circRNAs.
