# Supplementary material for: Hsa_circ_0015278 Regulates FLT3-ITD AML Progression via Ferroptosis-Related Genes
Source: Cancers (Basel). 2022 Dec 22;15(1):71. doi: 10.3390/cancers15010071 (PMC9817690; doi:10.3390/cancers15010071)
Supplement: Supplementary file 1 [file cancers-15-00071-s001.zip › Table S4. Details of 11 co-expressed circRNAs in GSE94591.pdf]

Table S4. Details of 11 co-expressed circRNAs in GSE94591

circBase ID	Gene Name	Location	AML & HC Log FC	$FLT3^{+}$ & $FLT3^{-}$ Log FC
hsa_circ_0049356	CARM1	chr19:10913876-10916777	4.222439	5.0956321
hsa_circ_0078051	AIG1	chr6:143486218-143605362	4.108214	4.2193528
hsa_circ_0011929	CTPS	chr1:40997393-41001117	4.074232	4.4832568
hsa_circ_0000370	FLI1	chr11:128758114-128782023	3.985417	5.5226337
hsa_circ_0011536	ZMYM4	chr1:35358924-35361789	3.664993	4.2394041
hsa_circ_0000086	ST6GALNAC3	chr1:76228473-76228588	3.621135	4.0861721
hsa_circ_0060828	TMEM189-UBE2V1	chr20:50127974-50143621	3.355358	3.6351438
hsa_circ_0003162	BBS9	chr7:33146241-33177591	3.11789	2.9581438
hsa_circ_0003568	IST1	chr16:71920738-71921453	2.993501	3.4411785
hsa_circ_0061817	C2CD2	chr21:41914597-41922085	2.97464	3.170246
hsa_circ_0015278	KLHL20	chr1:173756975-173775842	2.801769	3.1197386
