# Supplementary material for: Hsa_circ_0015278 Regulates FLT3-ITD AML Progression via Ferroptosis-Related Genes
Source: Cancers (Basel). 2022 Dec 22;15(1):71. doi: 10.3390/cancers15010071 (PMC9817690; doi:10.3390/cancers15010071)
Supplement: Supplementary file 1 [file cancers-15-00071-s001.zip › Table S5. GO and KEGG enrichment analysis of 73 FerRGs.pdf]

Table S5. GO and KEGG enrichment analysis of 73 FerRGs

| ID | Description | Count | p-value | FDR | mRNAs |
|---|---|---|---|---|---|
| **BP terms** | | | | | |
| GO:0010038 | response to metal ion | 14 | 2.06E-10 | 7.69E-08 | ATF4, CDH1, NQO1, G6PD, MTF1, NEDD4, NFE2L2, C11A2, MAPK3, MAPK8, TFR2, TXNIP, NEDD4L, SLC40A1 |
| GO:0034599 | cellular response to oxidative stress | 14 | 1.73E-11 | 1.84E-08 | ATF4, NQO1, FANCD2, G6PD, MYB, NCF2, NFE2L2, PRDX1, MAPK3, MAPK8, TSC1, ATG7, SIRT1, SRXN1 |
| GO:0031668 | cellular response to extracellular stimulus | 13 | 5.60E-11 | 2.93E-08 | ATF4, CDKN1A, LAMP2, NFE2L2, MAPK3, MAPK8, TSC1, VDR, ATG7, SIRT1, GABARAPL1, WIPI1, EIF2AK4 |
| GO:0072593 | reactive oxygen species metabolic process | 9 | 4.59E-06 | 4.29E-04 | NQO1, MYB, NFE2L2, PRDX1, MAPK3, MAPK8, TXNIP, SIRT1 |
| GO:0030099 | myeloid cell differentiation | 8 | 2.85E-04 | 7.04E-03 | ACVR1B, G6PD, SLC11A2, PRKCA, STAT3, TFRC, PIR, SIRT1 |
| GO:1901568 | fatty acid derivative metabolic process | 6 | 6.10E-05 | 2.75E-03 | ALOX12, AKR1C2, MAPK3, SCD, SIRT1, FAR1 |
| GO:0000302 | response to reactive oxygen species | 8 | 4.59E-06 | 4.29E-04 | NQO1, MYB, NFE2L2, PRDX1, MAPK3, MAPK8, TXNIP, SIRT1 |
| GO:0071248 | cellular response to metal ion | 8 | 1.04E-06 | 1.36E-04 | ATF4, CDH1, NQO1, NFE2L2, MAPK3, MAPK8, TFR2, SLC40A1 |
| GO:0002262 | myeloid cell homeostasis | 6 | 2.99E-05 | 1.63E-03 | ACVR1B, G6PD, HMGB1, SLC11A2, PRDX1, STAT3 |
| GO:0006826 | iron ion transport | 6 | 2.31E-07 | 3.78E-05 | FTL, SLC11A2, TFR2, TFRC, SLC39A14, SLC40A1 |
| **CC terms** | | | | | |
| GO:0031252 | cell leading edge | 7 | 9.95E-04 | 2.64E-02 | ATF4, CAPG, CD44, CDH1, TSC1, SLC39A14, GABARAPL1 |
| GO:0005770 | late endosome | 5 | 3.28E-03 | 7.67E-02 | LAMP2, SLC11A2, MAPK3, SQSTM1, NEDD4L |

| | | | | | |
|---|---|---|---|---|---|
| GO:0030027 | lamellipodium | 5 | 9.53E-04 | 2.64E-02 | CAPG, CD44, CDH1, TSC1, SLC39A14 |
| GO:0101002 | ficolin-1-rich granule | 5 | 7.88E-04 | 2.64E-02 | CTSB, HMGB1, LAMP2, SLC2A3, ATG7 |
| GO:0048770 | pigment granule | 5 | 5.93E-05 | 3.55E-03 | CAPG, CTSB, PRDX1, SLC3A2, TFRC |
| GO:0042470 | melanosome | 5 | 5.93E-05 | 3.55E-03 | CAPG, CTSB, PRDX1, SLC3A2, TFRC |
| GO:0005776 | autophagosome | 5 | 3.17E-05 | 3.55E-03 | FTL, LAMP2, SQSTM1, GABARAPL1, WIPI1 |
| GO:0005767 | secondary lysosome | 4 | 2.09E-07 | 4.99E-05 | FTL, LAMP2, NCF2, SQSTM1 |
| GO:0000421 | autophagosome membrane | 3 | 3.73E-04 | 1.48E-02 | LAMP2, GABARAPL1, WIPI1 |
| GO:0000407 | phagophore assembly site | 3 | 2.62E-04 | 1.25E-02 | SQSTM1, ATG7, WIPI1 |
| **MF terms** | | | | | |
| GO:0004674 | protein serine/threonine kinase activity | 9 | 1.19E-04 | 6.43E-03 | ACVR1B, GSK3B, MAP3K11, PRKCA, MAPK3, MAPK8, AURKA, SQSTM1, EIF2AK4 |
| GO:0031625 | ubiquitin protein ligase binding | 9 | 4.58E-06 | 1.21E-03 | ACVR1B, BID, CDKN1A, GSK3B, AURKA, SQSTM1, TXNIP, GABARAPL1, TRIB3 |
| GO:0050662 | coenzyme binding | 7 | 2.72E-04 | 8.84E-03 | DECR1, G6PD, PGD, POR, NFS1, SIRT1, CYB5R1 |
| GO:0051427 | hormone receptor binding | 5 | 1.27E-03 | 2.58E-02 | STAT3, VDR, NCOA3, SIRT1, WIPI1 |
| GO:0035257 | nuclear hormone receptor binding | 5 | 5.26E-04 | 1.42E-02 | STAT3, VDR, NCOA3, SIRT1, WIPI1 |
| GO:0005506 | iron ion binding | 4 | 4.36E-03 | 5.06E-02 | ALOX12, FTL, RRM2, SCD |
| GO:0050661 | NADP binding | 4 | 7.65E-05 | 6.22E-03 | DECR1, G6PD, PGD, POR |
| GO:0016209 | antioxidant activity | 3 | 6.26E-03 | 5.81E-02 | NQO1, PRDX1, SRXN1 |
| GO:0046915 | transition metal ion transmembrane | 3 | 5.10E-04 | 1.42E-02 | SLC11A2, SLC39A14, SLC40A1 |

| | | | | | |
|---|---|---|---|---|---|
| | transporter activity | | | | |
| GO:0008199 | ferric iron binding | 2 | 1.00E-03 | 2.33E-02 | FTL, RRM2 |
| **KEGG** | | | | | |
| hsa04216 | Ferroptosis | 8 | 3.41E-10 | 6.93E-08 | FTL, SLC11A2, SAT1, SLC3A2, TFRC, ATG7, SLC39A14, SLC40A1 |
| hsa04218 | Cellular senescence | 6 | 8.34E-04 | 9.03E-03 | CDKN1A, CDKN2A, MAPK3, TSC1, SQSTM1, SIRT1 |
| hsa04068 | FoxO signaling pathway | 6 | 3.29E-04 | 5.74E-03 | CDKN1A, MAPK3, MAPK8, STAT3, SIRT1, GABARAPL1 |
| hsa04150 | mTOR signaling pathway | 5 | 4.93E-03 | 2.38E-02 | GSK3B, PRKCA, MAPK3, SLC3A2, TSC1 |
| hsa04066 | HIF-1 signaling pathway | 5 | 1.06E-03 | 9.78E-03 | CDKN1A, PRKCA, MAPK3, STAT3, TFRC |
| hsa04012 | ErbB signaling pathway | 5 | 3.39E-04 | 5.74E-03 | CDKN1A, GSK3B, PRKCA, MAPK3, MAPK8 |
| hsa04115 | p53 signaling pathway | 5 | 1.66E-04 | 4.07E-03 | BID, CDKN1A, CDKN2A, CD82, RRM2 |
| hsa04912 | GnRH signaling pathway | 4 | 4.35E-03 | 2.21E-02 | ATF4, PRKCA, MAPK3, MAPK8 |
| hsa04978 | Mineral absorption | 4 | 8.12E-04 | 9.03E-03 | FTL, SLC11A2, VDR, SLC40A1 |
| hsa00480 | Glutathione metabolism | 3 | 7.82E-03 | 3.31E-02 | G6PD, PGD, RRM2 |
