# Supplementary material for: Hsa_circ_0015278 Regulates FLT3-ITD AML Progression via Ferroptosis-Related Genes
Source: Cancers (Basel). 2022 Dec 22;15(1):71. doi: 10.3390/cancers15010071 (PMC9817690; doi:10.3390/cancers15010071)
Supplement: Supplementary file 1 [file cancers-15-00071-s001.zip › Table S6. 15 hub genes were obtained by the 12 algorithms.pdf]

Table S6. 15 hub genes were obtained by the 12 algorithms

	SIRT1	MAPK3	MAPK8	NFE2L2	CDKN1A	CDH1	CDKN2A	STAT3
**MCC**	5567.0	5580.0	5289.0	5700.0	5308.0	5356.0	5162.0	5669.0
**DMNC**	0.38858	0.46068	0.54289	0.31531	0.63848	0.61085	0.62053	0.45768
**MNC**	17.0	15.0	11.0	24.0	10.0	11.0	9.0	16.0
**Degree**	18.0	17.0	12.0	24.0	10.0	11.0	9.0	17.0
**EPC**	20.809	20.499	19.218	21.815	18.342	18.873	18.105	21.042
**BottleNeck**	12.0	8.0	2.0	14.0	3.0	3.0	1.0	6.0
**EcCentricity**	0.25	0.25	0.25	0.25	0.25	0.25	0.25	0.25
**Closeness**	37.75	37.83333	34.1667	41.83333	33.08333	33.33333	32.58333	37.83333
**Radiality**	6.07813	6.125	5.92188	6.28125	5.89063	5.875	5.875	6.125
**Betweenness**	400.13101	405.15776	132.5647	989.55028	59.45558	63.89781	58.03249	371.11543
**Stress**	1568.0	1452.0	492.0	3008.0	402.0	338.0	392.0	1272.0
**ClusteringCoefficient**	0.31373	0.33824	0.48485	0.25362	0.71111	0.65455	0.72222	0.375

	ATG7	TFRC	SQSTM1	CD44	GSK3B	G6PD	SRXN1
**MCC**	416.0	31.0	204.0	152.0	219.0	16.0	21.0
**DMNC**	0.42441	0.56839	0.31046	0.38186	0.45891	0.47366	0.38039
**MNC**	12.0	4.0	15.0	9.0	10.0	4.0	6.0
**Degree**	12.0	10.0	15.0	11.0	11.0	8.0	7.0
**EPC**	18.717	13.119	19.394	17.45	18.001	10.257	12.455
**BottleNeck**	4.0	9.0	7.0	3.0	1.0	4.0	3.0
**EcCentricity**	0.25	0.2	0.25	0.2	0.25	0.25	0.25
**Closeness**	34.25	33.23333	36.41667	32.48333	33.58333	30.91667	31.0
**Radiality**	5.92188	5.85938	6.0625	5.75	5.90625	5.73438	5.78125
**Betweenness**	134.53345	448.24402	393.71901	255.81264	129.03965	339.69365	293.53975
**Stress**	612.0	1142.0	1506.0	964.0	584.0	750.0	714.0
**ClusteringCoefficient**	0.43939	0.17778	0.29524	0.29091	0.41818	0.21429	0.38095
