# Supplementary material for: Hsa_circ_0015278 Regulates FLT3-ITD AML Progression via Ferroptosis-Related Genes
Source: Cancers (Basel). 2022 Dec 22;15(1):71. doi: 10.3390/cancers15010071 (PMC9817690; doi:10.3390/cancers15010071)
Supplement: Supplementary file 1 [file cancers-15-00071-s001.zip › Table S7 Summary of hub genes regulating AML progression.pdf]

Table S7 Summary of hub genes regulating AML progression

| hub gene | Official Full Name (HGNC) | Effects on AML Progression | PMID |
|---|---|---|---|
| SIRT1 | Sirtuin 1 | SIRT regulated by FLT3 kinase activity plays a major role in harboring activating mutations. | 24855208 |
| | | Inhibition of SIRT1 expression or activity reduced the growth of FLT3-ITD AML LSCs. | 25280219 |
| NFE2L2 | NFE2 Like BZIP Transcription Factor 2 | High NFE2L2 expression can induce gene instability-dependent drug resistance in AML. | 33414469 |
| CDKN1A | Cyclin Dependent Kinase Inhibitor 1A | CDKN1A promotes G0-G1 cell-cycle arrest, and triggers apoptosis in AML cells. | 29626127 |
| CDKN2A | Cyclin Dependent Kinase Inhibitor 2A | Reduced expression of CDKN2A conferred inferior overall survival in human AML cohorts. | 36400926 |
| CDH1 | Cadherin 1 | Promoter hypermethylation of the CDH1 genes was closely associated with an internal tandem duplication (ITD) of the FLT3 gene. | 16281935 |
| STAT3 | Signal Transducer And Activator Of Transcription 3 | STAT pathways could be potential targets for reducing resistance developed in AML patients receiving FLT3 inhibitors. | 19144991 |
| ATG7 | Autophagy Related 7 | The key autophagy genes Atg5 and Atg7 reversed the FLT3 degradation to regulate the progression of FLT3-ITD mutated AML. | 32284743 |
| TFRC | Transferrin Receptor | TFRC/$Fe2^{+}$ pathway exhibits potent anti-AML activity. | 36368726 |
| SQSTM1 | Sequestosome 1 | p62/SQSTM1 delivered FLT3-ITD proteins to the lysosome for subsequent degradation which may affect FLT3-ITD AML progression. | 32284743 |

| | | | |
|---|---|---|---|
| CD44 | CD44 Molecule (Indian Blood Group) | CD44 plays a major role in AML patients harboring FLT3 mutations. | 36163632 |
| GSK3B | Glycogen Synthase Kinase 3 Beta | Phosphorylation of ERK and GSK3B regulated high-risk AML patients with FLT3 wildtype. | 35857899 |
| G6PD | Glucose-6-Phosphate Dehydrogenase | Inactivating ATM or its downstream effector G6PD sensitizes AML cells to FLT3 inhibitor-induced apoptosis. | 27791036 |
