# Supplementary figures and images for: Hsa_circ_0015278 Regulates FLT3-ITD AML Progression via Ferroptosis-Related Genes
Source: Cancers (Basel). 2022 Dec 22;15(1):71. doi: 10.3390/cancers15010071 (PMC9817690; doi:10.3390/cancers15010071)

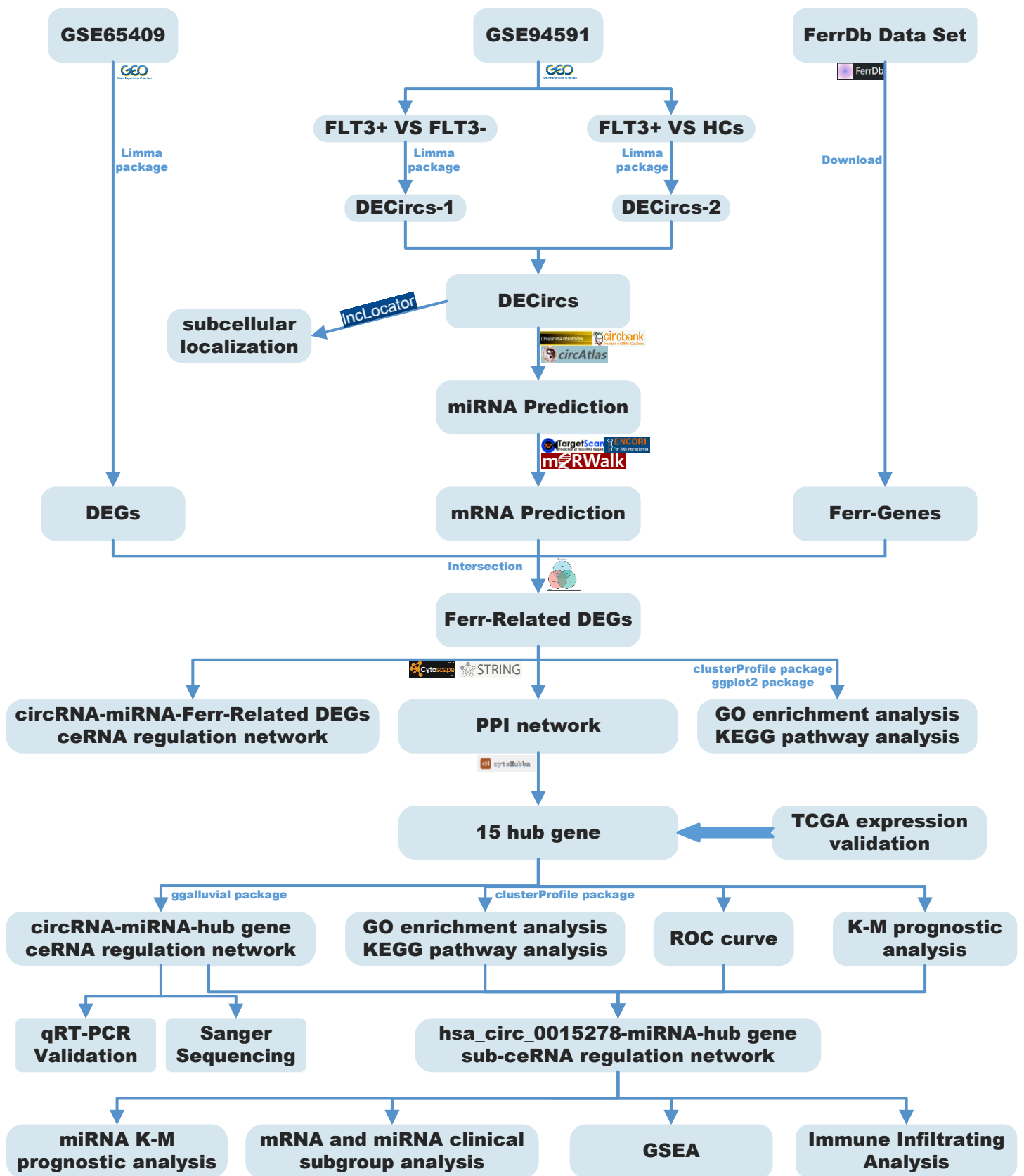

Supplement: Supplementary file 1 [file cancers-15-00071-s001.zip › Fig.S1 Detailed flowchart of the comprehensive analysis and methods utilized in the present study.pdf]

**A**

Volcano plot  
GSE65409 AML VS HCs

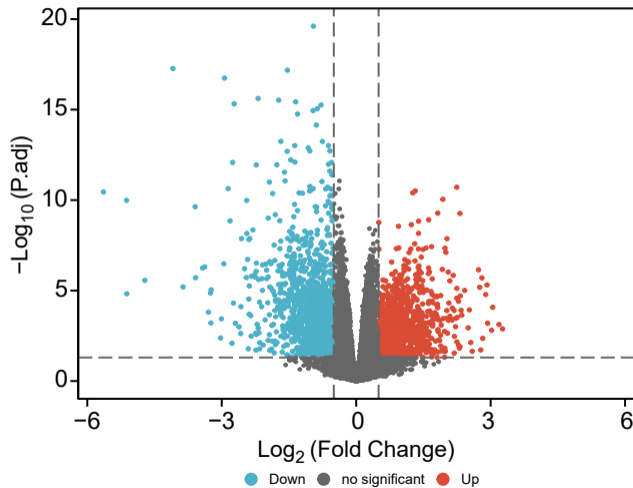**B**

Heatmap plot  
GSE65409 AML VS HCs

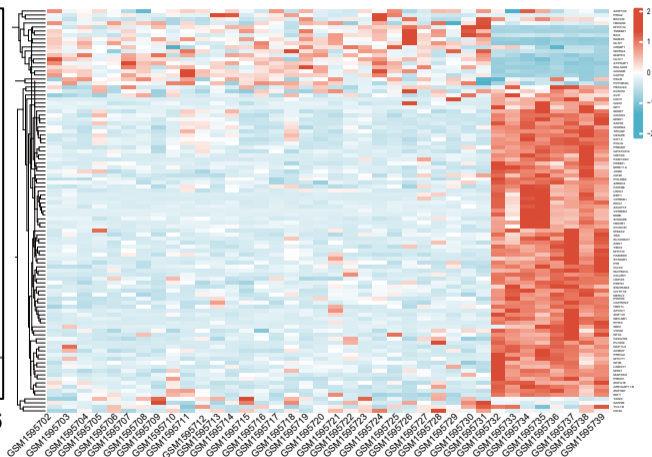

Supplement: Supplementary file 1 [file cancers-15-00071-s001.zip › Fig.S2 Identification of DEGs in AML.pdf]

A

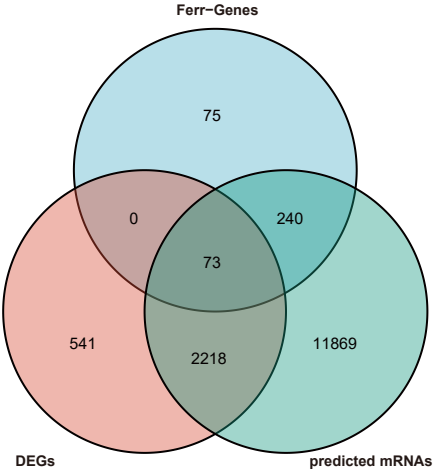

B

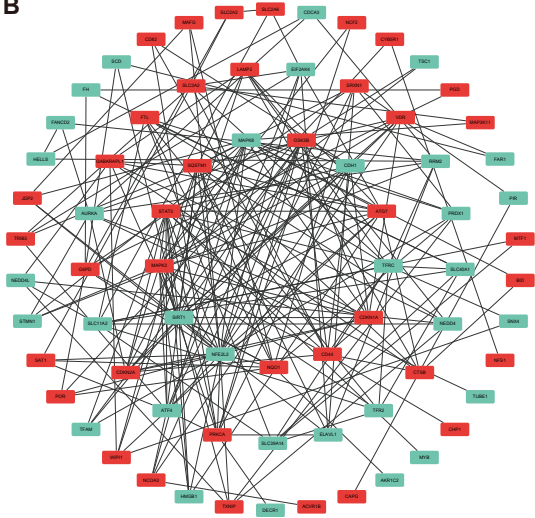

Supplement: Supplementary file 1 [file cancers-15-00071-s001.zip › Fig.S3 73 FerRGs were presented via Venn Diagram and PPI network.pdf]

A

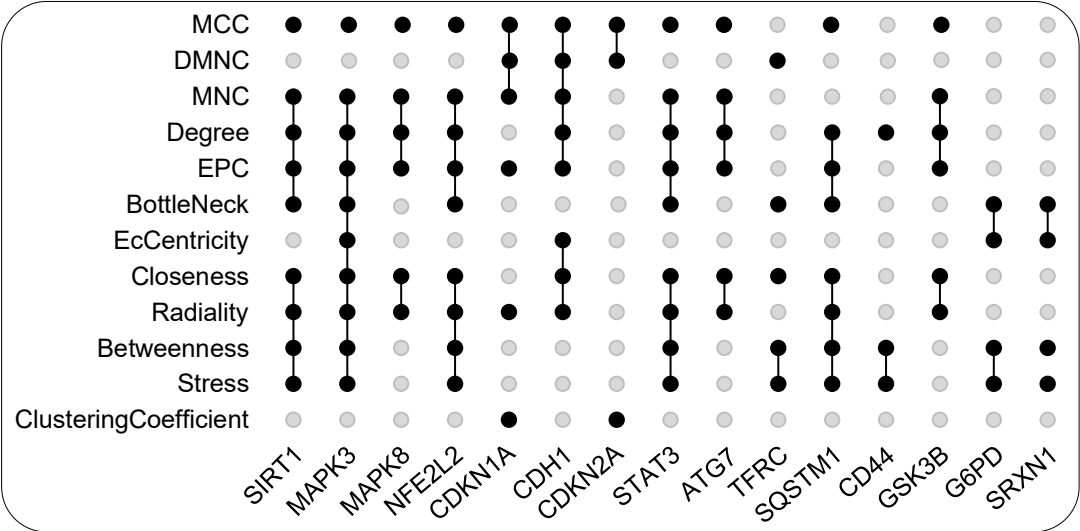

B

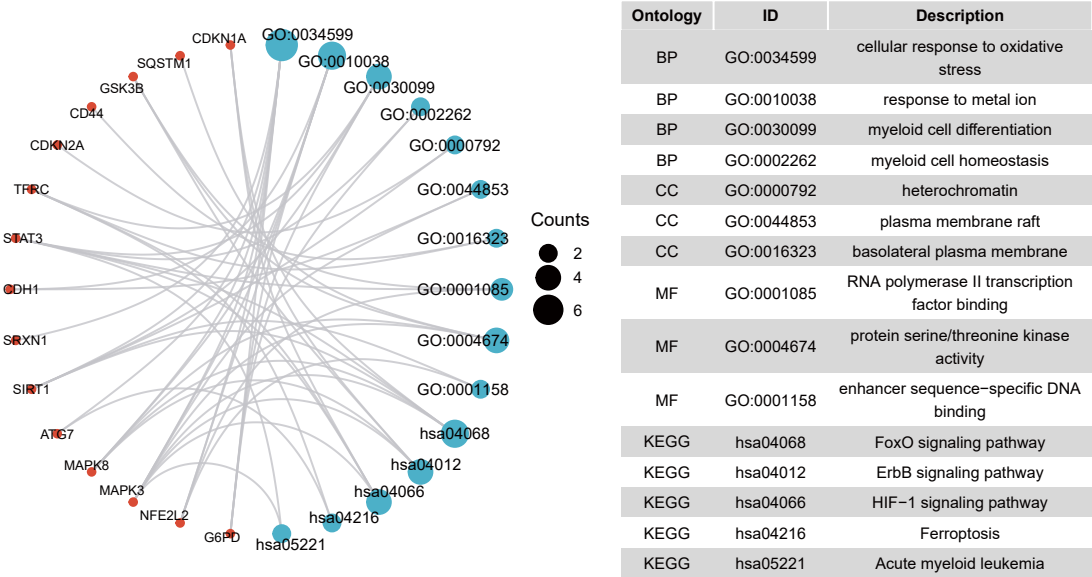

Supplement: Supplementary file 1 [file cancers-15-00071-s001.zip › Fig.S4 Screening and functional enrichment analysis of 15 hub genes.pdf]

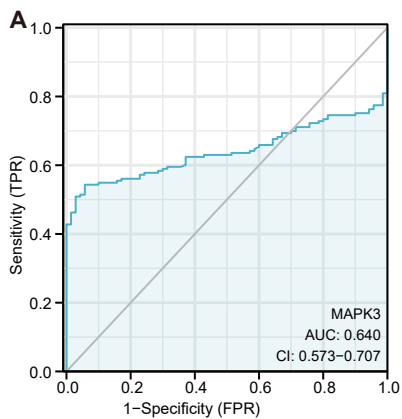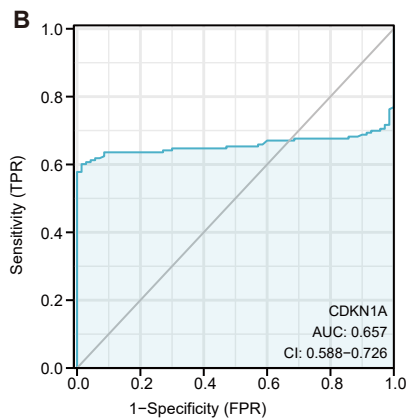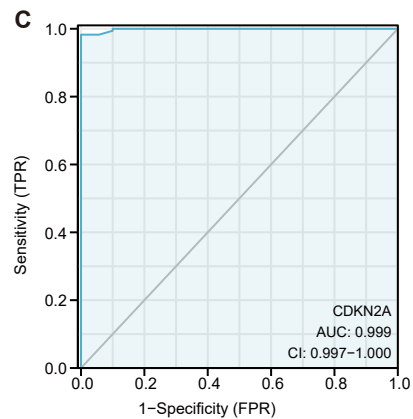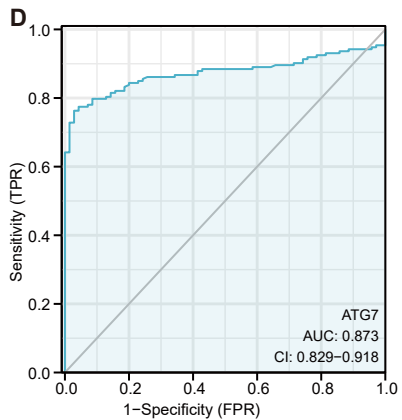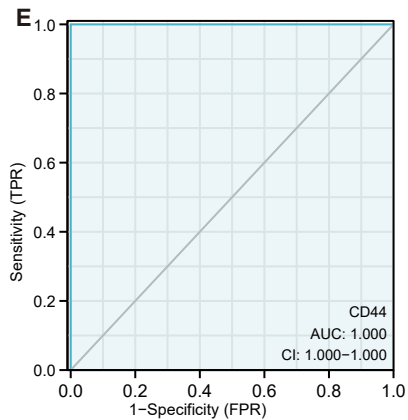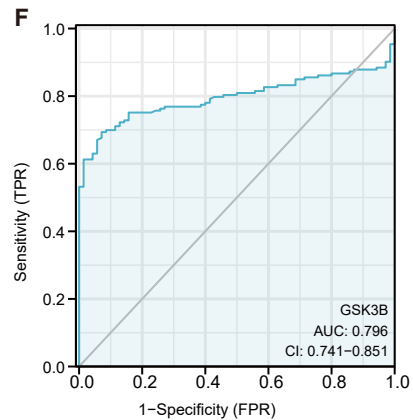

Supplement: Supplementary file 1 [file cancers-15-00071-s001.zip › Fig.S5 Evaluation of the diagnostic efficacy of hub genes.pdf]

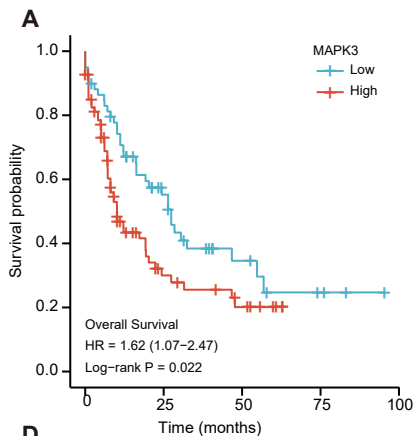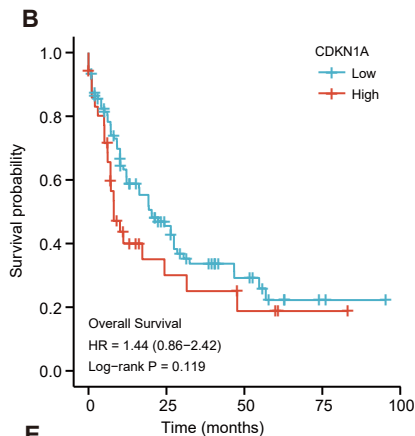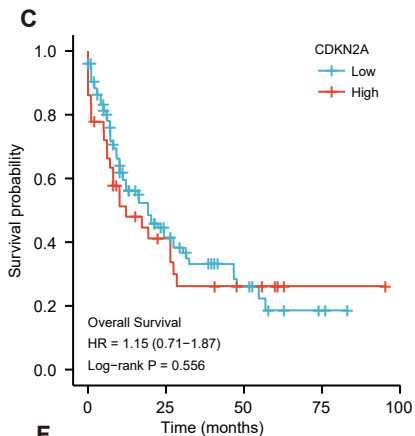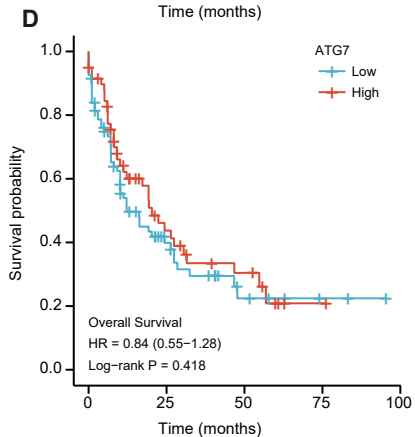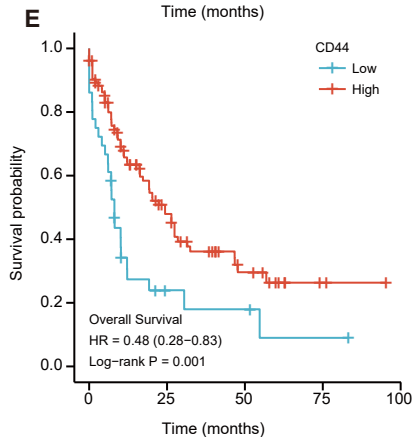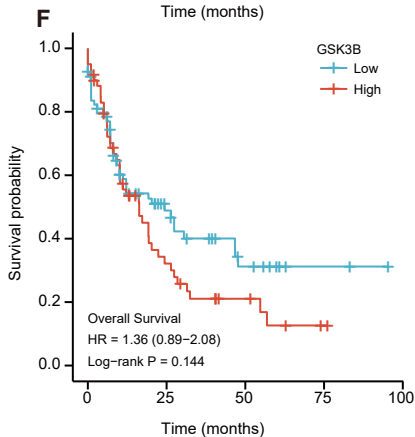

Supplement: Supplementary file 1 [file cancers-15-00071-s001.zip › Fig.S6 Evaluation of the prognostic efficacy of hub genes.pdf]

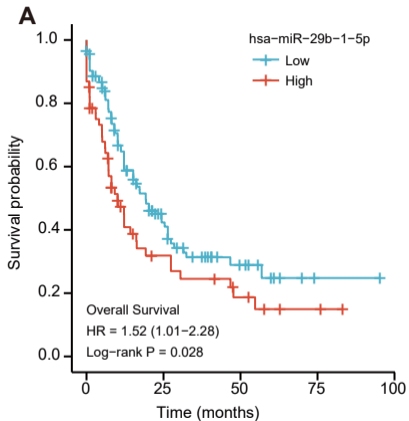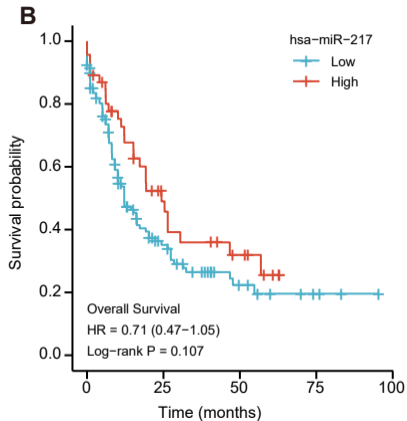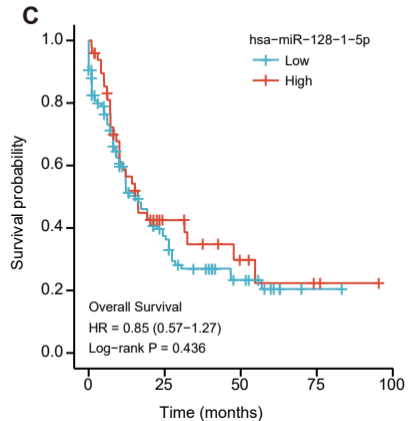

Supplement: Supplementary file 1 [file cancers-15-00071-s001.zip › Fig.S7 Evaluation of the prognostic efficacy of miRNAs.pdf]

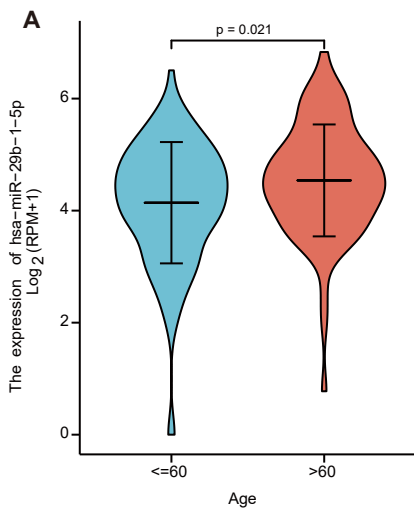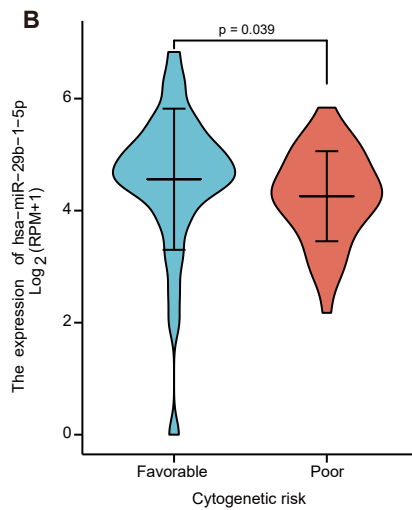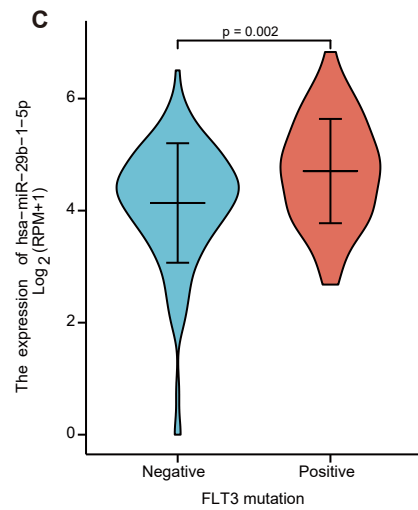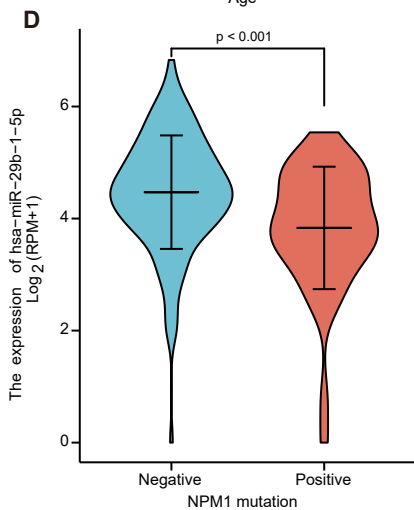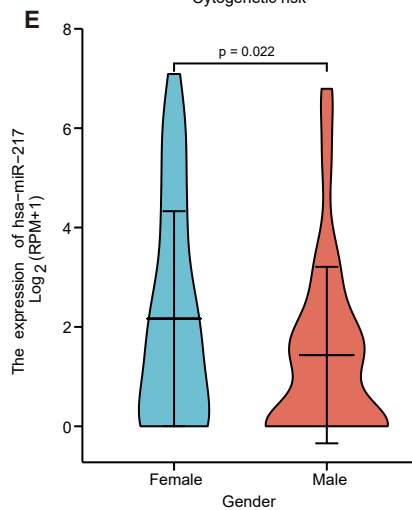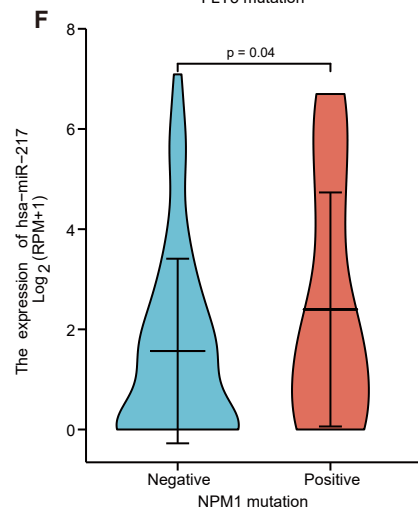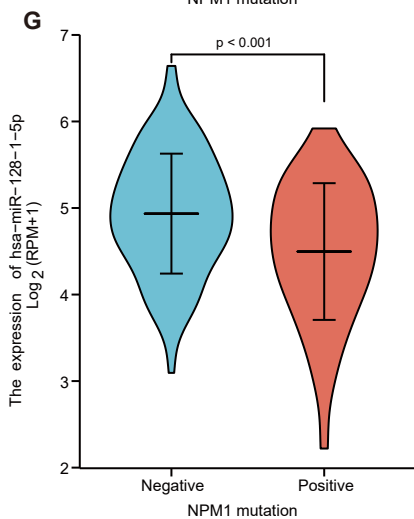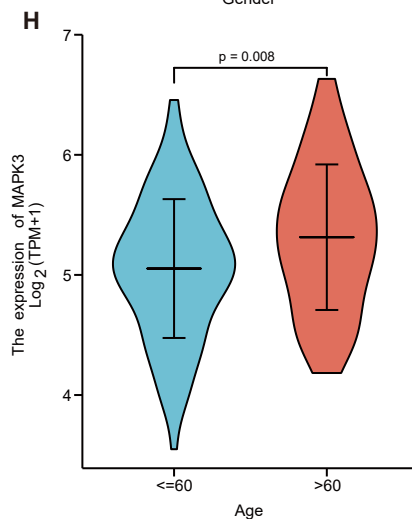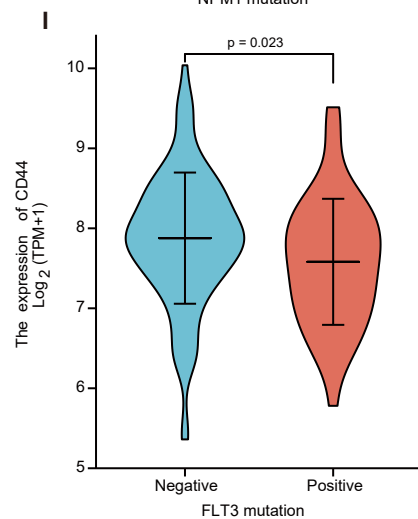

Supplement: Supplementary file 1 [file cancers-15-00071-s001.zip › Fig.S8 Correlation analysis between miRNAs and mRNAs involved in the sub-network and clinicopathological parameters.pdf]

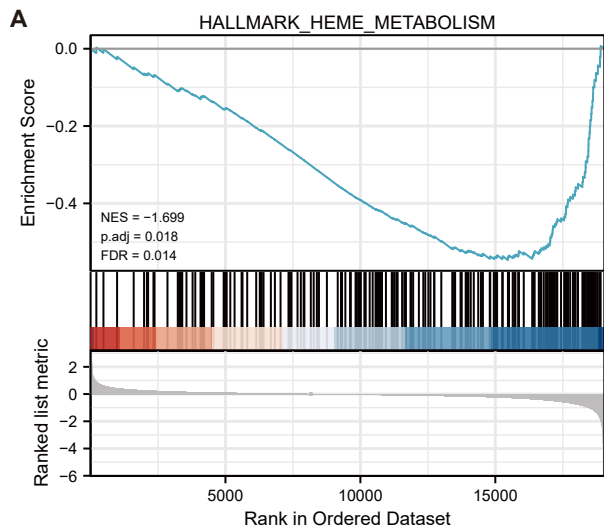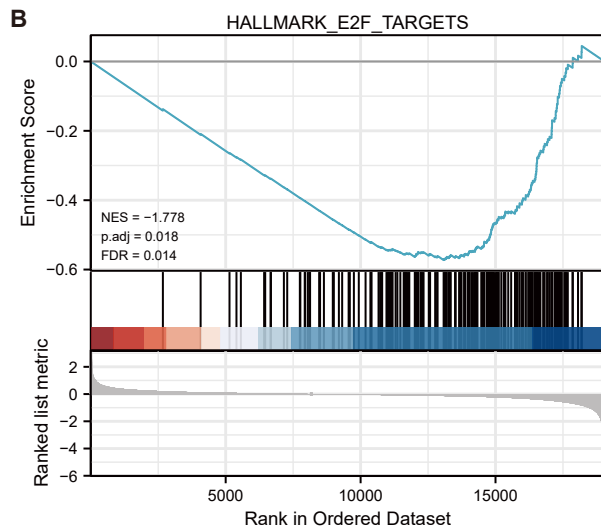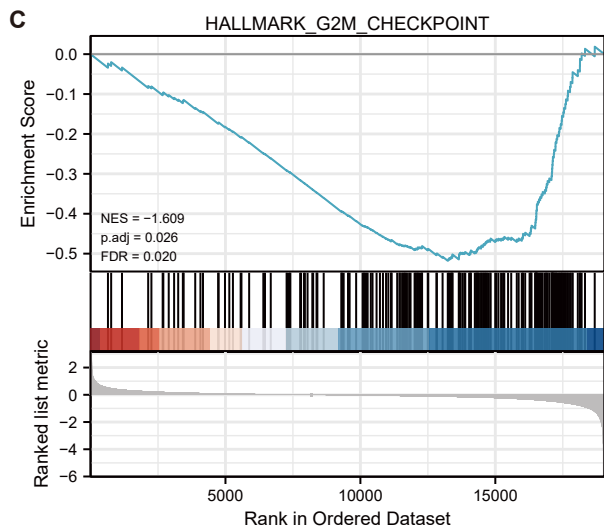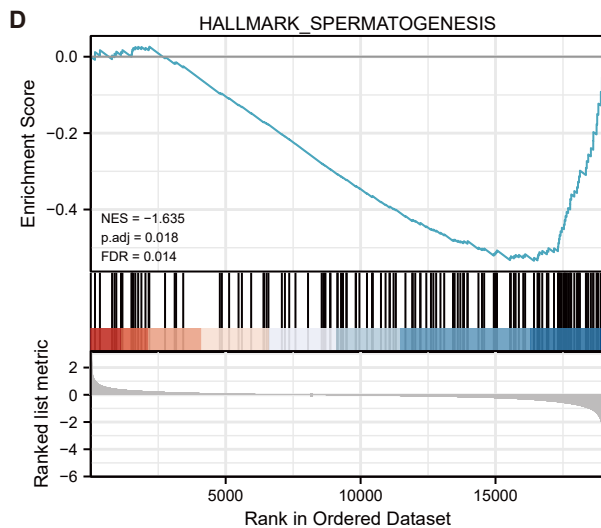

Supplement: Supplementary file 1 [file cancers-15-00071-s001.zip › Fig.S9 GSEA enrichment analysis of CD44.pdf]
